# Results of Endoscopic Treatment for Early Gastric Cancer by Nd-YAG Laser

**DOI:** 10.1155/DTE.3.203

**Published:** 1997

**Authors:** M. Tani, K. Takeshita, T. Honda, N. Saito, M. Endo

**Affiliations:** 1 First Department of Surgery Tokyo Medical and Dental University School of Medicine 1-5-45, Bunkyo-ku, Yushima Tokyo Tokyo 113 Japan; 2 Department of Endoscopic Diagnosis and Therapy Tokyo Medical and Dental University School of Medicine Tokyo Tokyo 113 Japan

## Abstract

We have introduced two endoscopic treatments for early gastric cancer: endoscopic mucosal resection using a cap-fitted panendoscope (EMRC), and endoscopic laser therapy using a Nd-YAG laser. Thirty-two patients (34 lesions) with gastric cancer were treated by Nd-YAG laser; including 23 initial-therapy cases (25 lesions) and 9 second-therapy cases representing failures of endoscopic mucosal resection or endoscopic ethanol injection. Endoscopic laser therapy was performed safely without complication in all patients. Three patients had residual cancer, and 2 of these required surgery. Six patients died from other disease. Endoscopic laser therapy can remove early gastric cancer even when the lesion has ulceration or submucosal invasion, and has a powerful hemostatic effect. It is a safe and effective treatment for early gastric cancer.


*Diagnostic and Therapeutic Endoscopy*, 1997, Vol. 3, pp. 203-210
Reprints available directly from the publisher
Photocopying permitted by license only

© 1997 OPA (Overseas Publishers Association)

Amsterdam B.V. Published in The Netherlands by Harwood Academic Publishers

Printed in Singapore

### Results of Endoscopic Treatment for Early Gastric Cancer by Nd-YAG Laser

M. TANI[1]*, K. TAKESHITA[1,2], T. HONDA[1], N. SAITO[1] and M. ENDO[1]

[1]*First Department of Surgery, and* [2]*Department of Endoscopic Diagnosis and Therapy, Tokyo Medical and Dental University School of Medicine, Tokyo, Tokyo 113, Japan*

*(Received 26 August 1996; in final form 29 November 1996)*

**We have introduced two endoscopic treatments for early gastric cancer: endoscopic mucosal resection using a cap-fitted panendoscope (EMRC), and endoscopic laser therapy using a Nd-YAG laser. Thirty-two patients (34 lesions) with gastric cancer were treated by Nd-YAG laser, including 23 initial-therapy cases (25 lesions) and 9 second-therapy cases representing failures of endoscopic mucosal resection or endoscopic ethanol injection. Endoscopic laser therapy was performed safely without complication in all patients. Three patients had residual cancer, and 2 of these required surgery. Six patients died from other disease. Endoscopic laser therapy can remove early gastric cancer even when the lesion has ulceration or submucosal invasion, and has a powerful hemostatic effect. It is a safe and effective treatment for early gastric cancer.**

*Keywords*: early gastric cancer, endoscopic treatment, laser therapy, Nd-YAG laser

### INTRODUCTION

Neodymium yttrium aluminum garnet (Nd-YAG) laser treatment has been used to alleviate obstruction from unresectable malignant lesions of the gastrointestinal tract [1], to arrest hemorrhage from gastric vascular ectasia [2], and to treat early gastric cancer curatively [3].

Curative endoscopic treatment for early gastric cancer has become accepted at many institutions in Japan, incorporating such specific modalities as polypectomy, mucosal resection (EMR), laser, heatprobe, microwave, injections and hyperthermia. We have introduced two endoscopic treatments for early gastric cancer. The first is an endoscopic mucosal resection using a

*Address correspondence to: Masao Tani, First Department of Surgery, Tokyo Medical and Dental University, 1-5-45, Bunkyo-ku, Yushima, Tokyo 113 Japan Tel: 03-5803-5255; Fax: 03-3817-4126.

cap-fitted panendoscope (EMRC) [4–6]. The second is a laser therapy using a medical Nd-YAG laser system (MC2100) [7].

We examined our results with endoscopic laser therapy and evaluated its role in the endoscopic treatment of early gastric cancer.

### MATERIALS AND METHODS

### Indication

Absolute indications for endoscopic treatment of early gastric cancer were well-differentiated tubular adenocarcinoma and type 0'IIa smaller than 20 mm in diameter or type 0'IIb or 0'IIc smaller than 10 mm in diameter without ulcer or ulcer scar. Relative indications for treatment were inoperable tumors in patients who were poor surgical risks or who refused surgery. The macroscopic type of early gastric cancer was assessed according to the Japanese Classification of Gastric Carcinoma [8].

### Patients

Thirty-two patients with 34 lesions of early gastric tumor were treated by endoscopic Nd-YAG laser irradiation from August 1987 to October 1995. Of 32 cases of laser therapy, 23 cases (25 lesions) were initial-therapy cases. Nine cases were second-therapy cases, representing residual tumor following EMR (6 lesions) or endoscopic ethanol injection (3 lesions). Of 23 initial-therapy cases, 15 (16 lesions) were absolute indication lesions (adenoma: 4 lesions, type 0'IIa: 71 lesions, type 0'IIc: 5 lesions). Eight cases (9 lesions) were relative indication lesions (type 0'IIa: 2 lesions, type 0'I: 1 lesion, type 0'IIa + IIc: 1 lesion, type 0'IIc: 1 lesion, type 0'IIc + IIa: 4 lesions). Table 1 shows the characteristics of patients and lesions. There were 28 men, ranging in age from 58 to 84 (mean, 75) years and 4 women, ranging in age from 60 to 88 (mean, 76) years. In all cases, a histological diagnosis was obtained from examination of biopsy specimens. Location and macroscopic type were assessed by endoscopic findings. Follow-up period ranged from one to 98 months.

Table I Thirty-two cases of 34 gastric tumorous lesions were treated by Nd-YAG laser system MC2100 from August 1987 – September 1995.

| | | | | | |
|---|---|---|---|---|---|
| Male: Female | 28:4 | | | | |
| Mean age | Male: 75 (58~74) | | Female: 76 (60~88) | | |
| | | Lower | Middle | Upper | |
| Location | A.W. | 3 | 2 | 0 | 5 |
| | L.C. | 6 | 8 | 5 | 19 |
| | P.W. | 3 | 3 | 2 | 8 |
| | G.C. | 0 | 0 | 2 | 2 |
| | | 12 | 13 | 9 | 34 |
| Macroscopic type | adenoma | 4 | 0IIc | | 6 |
| | 0IIa | 9 | 0IIc+IIa | | 4 |
| | 0I | 1 | after EMR | | 6 |
| | 0IIa+IIc | 1 | after ethanol injection | | 3 |

A.W. anterior wall P.W. posterior wall

L.C. lesser curvature G.C. greater curvature

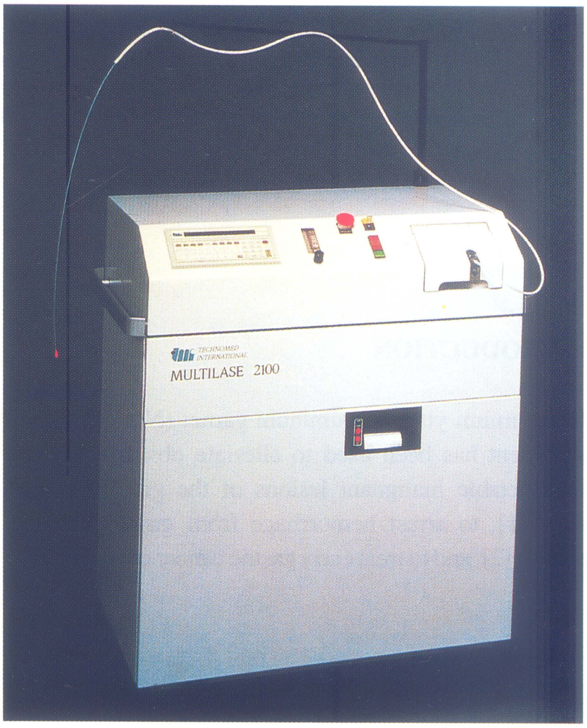

FIGURE 1 Medical Nd-YAG laser system MC2100.

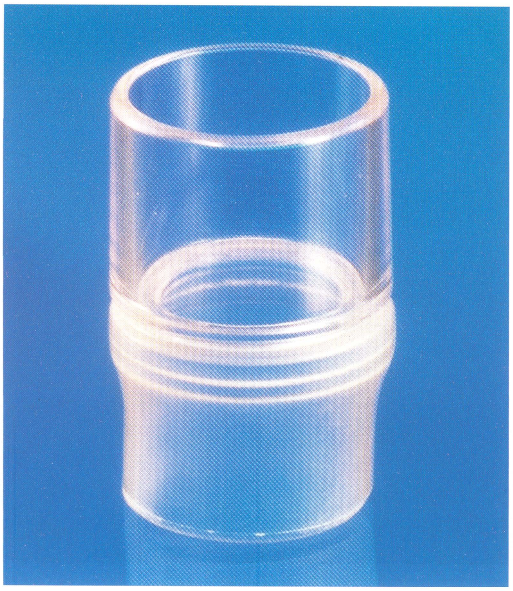

FIGURE 2a An EMRC cap (transparent plastic hood).

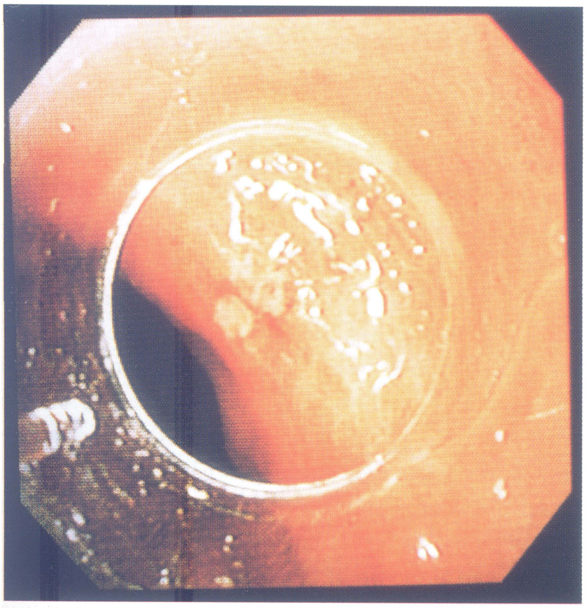

FIGURE 2b An EMRC cap (transparent plastic hood) is fitted to the tip of the panendoscope, and the laser fiber is passed through the biopsy channel.

### Methods

We used a medical YAG laser system MC2100 (Technomedical International Corp., France; Fig. 1) which has a 1.32-µm wavelength in addition to the conventional 1.06-µm wavelength. The 1.32-µm wavelength is more highly absorbed by living tissues than the 1.06-µm wavelength. Thus, endoscopic laser irradiation can be performed more safely and effectively with lower output using the 1.32-µm wavelength. In most cases we selected a 1.32-µm wavelength, 20 watts of output, continuous wave, and the non-contact method.

We performed laser irradiation using a transparent EMRC cap as Ozaki's method [9]. An EMRC cap (transparent plastic hood; Fig. 2a) was fitted to the tip of the endoscope and the laser fiber was passed through the biopsy channel (Fig. 2b). Using the cap, we obtained a good view in a variety of lesion sites and maintained the necessary distance between the laser fiber and the lesion during irradiation.

### Follow-up

We medicated all patients with $H_2$-blockers and sucralfate (mucosa-protecting drugs) for 1 month after treatment. Endoscopic examination and pathological examination of biopsy specimens were performed 1, 3, 6, 9 and 12 months after treatment, and then twice a year.

### RESULTS

Twenty-four in 32 patients were treated on an ambulatory basis with endoscopic laser therapy. Eight patients were treated as inpatients. Fifteen of the 34 lesions were laser-irradiated once, and 19 lesions were irradiated more than once. Two patients with a total of these lesions had laser therapy because EMR was contraindicated. One was a patient with a pacemaker for whom it was not safe to use high-frequency electrocautery, and another was a hemodialysis patient with liver cirrhosis and hypocoagulability.

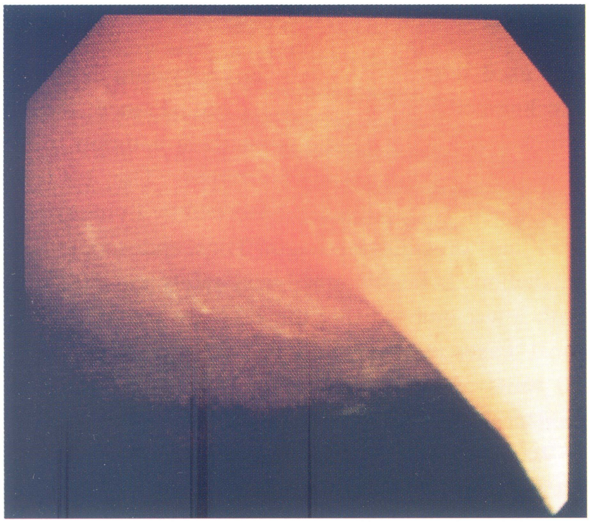

FIGURE 3a Residual cancer after endoscopic mucosal resection located on the lesser curvature of the upper body of the stomach is detected in the biopsy specimen from the small reddish area of the scar.

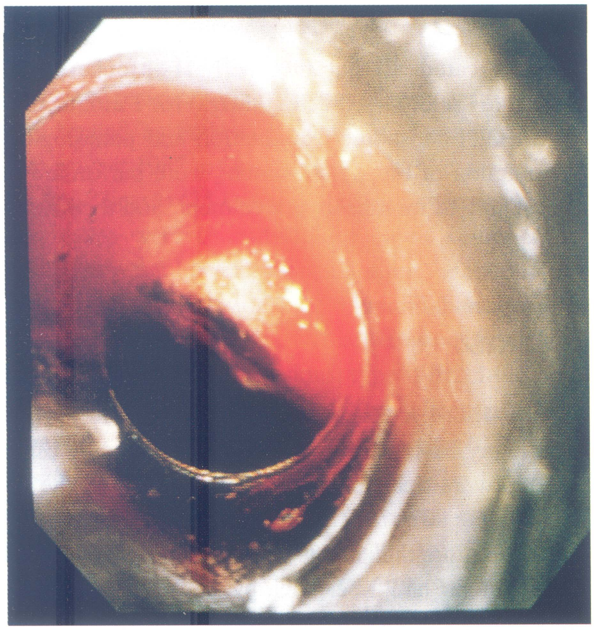

FIGURE 3b Laser irradiation is performed using an EMRC cap with the MC2100.

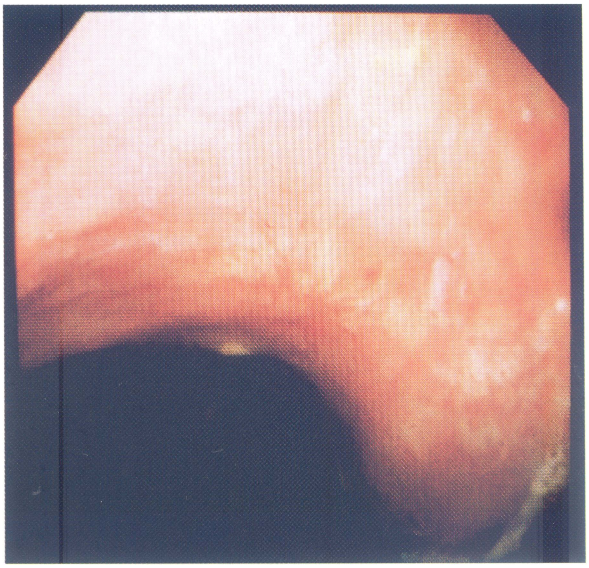

FIGURE 3c Endoscopic examination performed 6 months after treatment shows scar without residual disease.

### Complications

We experienced no complications in 32 cases of endoscopic laser therapy using the medical YAG laser system MC2100.

### Illustrative Case

Figure 3 shows a case of residual cancer following EMRC in a 72-year-old man. A biopsy specimen from a small reddish area of the edge of the scar revealed adenocarcinoma (Fig. 3a). We performed endoscopic laser irradiation using an EMRC cap with the MC2100 (non-contact method, continuous wave, 1.32-µm wavelength, 20 watts, and 510 joules). Because the lesion was located in the upper gastric body on the lesser curvature, only an oblique view of the lesion was obtained by usual endoscopic examination. The EMRC cap at the tip of the endoscope allowed a front view of the lesion, and vertical laser irradiation was performed (Fig. 3b). The patient was cancer-free 27 months following laser therapy (Fig. 3c).

Table II Outcome of 32 cases of 34 gastric tumorous lesions treated by Nd-YAG laser therapy

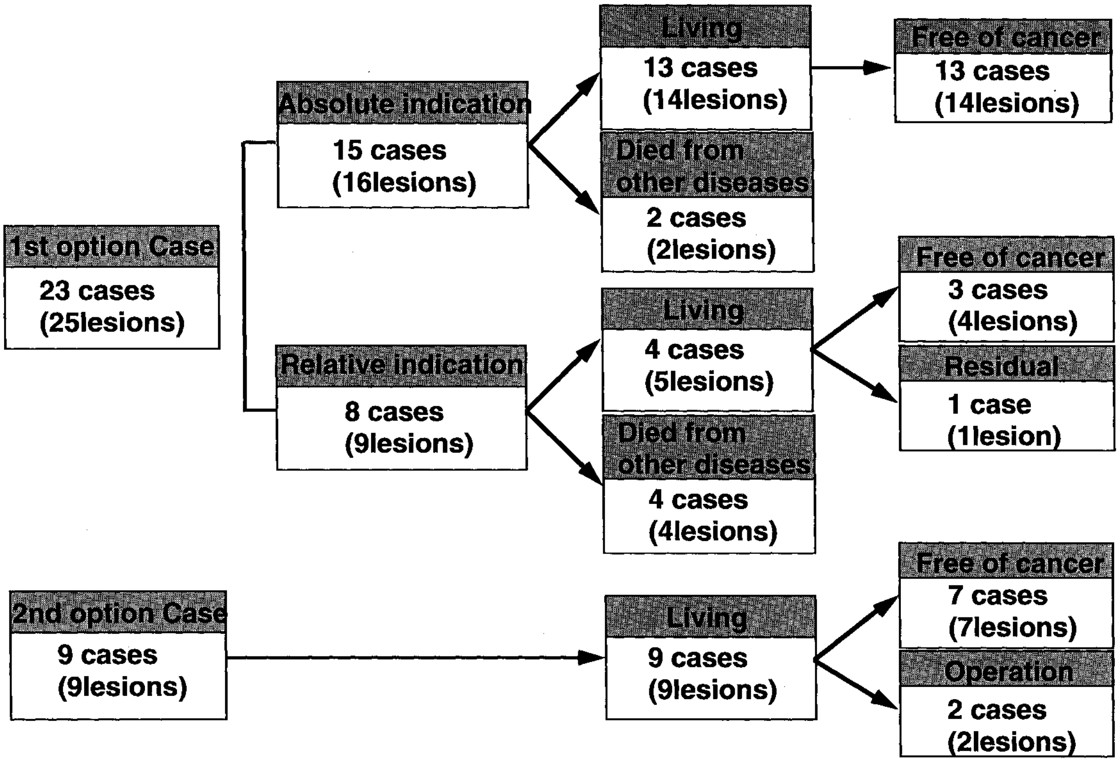

### Outcome

Table II shows the outcome of 32 cases (34 lesions) of gastric tumor treated by endoscopic laser therapy using the medical YAG-laser system MC2100. Twenty-six patients are living, of which 23 are cancer-free. Of three patients with residual cancer, two required surgery and one is undergoing additional laser therapy at the time of this writing. Six patients died from intercurrent disease.

One of the two operative cases was an 80-year-old man with residual cancer following EMR at another center. Cancer remained following five treatments with laser therapy; we therefore performed a gastric resection. Macroscopic findings in the resected stomach were a small tumor in the whitish area (Fig. 4a).

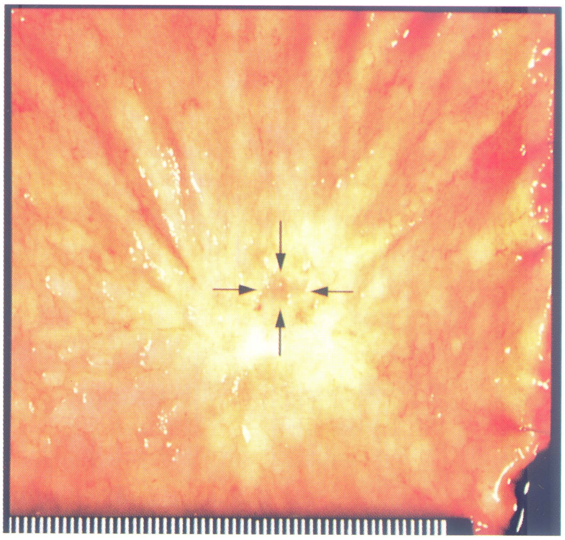

FIGURE 4a Resected stomach.

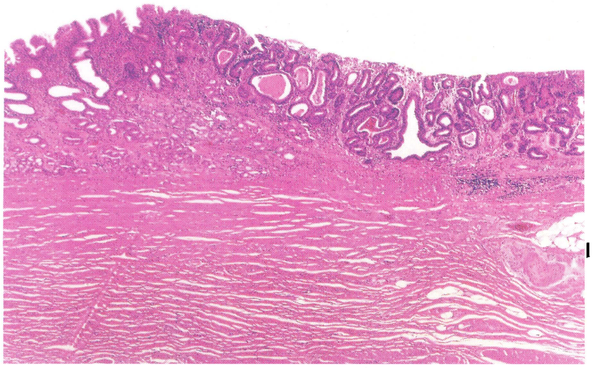

Histological examination of the irradiated area revealed cancer cells remained in the mucosal layer. Almost all of the submucosa was replaced by fibrosis (Fig. 4b).

### DISCUSSION

Endoscopic laser therapy has several roles in the treatment of gastric cancer including alleviation of stenosis in advanced cases, hemostasis, and curative treatment in early disease.

Gastric mucosal cancers rarely demonstrate lymph node metastasis, and it is widely accepted in Japan that small gastric mucosal cancers without lymph node metastasis can be curatively treated endoscopically. Single early gastric cancers resected at our department were retrospectively analyzed with regard to the depth of invasion and the incidence of lymph node metastasis [10]. From this we determined our absolute indications for endoscopic treatment. Curative treatment is possible for lesions of well-differentiated tubular adeno carcinoma and type 0IIa smaller than 20 mm or type 0IIb or 0IIc smaller than 10 mm without ulcer or ulcer scar. Relative indications for treatment are inoperable tumors secondary to poor risk factors or refusal of surgery.

Endoscopic mucosal resection (EMR) allows more accurate histopathological analysis than other endoscopic treatments. Using our modified EMR procedure (EMRC), large specimens can be obtained and lesions that are located on the lesser curvature or posterior wall of the body of the stomach, which are difficult to resect by conventional EMR, can be resected completely with ease.

Fifty-seven patients with 68 lesions of early gastric tumor were treated by EMRC procedure from August 1992 to October 1995. In 57 cases of EMRC, we experienced 3 cases of muscular resection and 5 cases of bleeding. Of 57 patients, 56 are living. One died from intercurrent disease. Two patients had residual tumor and required additional laser therapy. Two patients required surgery. Fifty-four of our 57 patients having EMRC were treated as inpatients.

Safe and sufficient mucosal resection cannot be performed when the lesion has fibrosis from an ulcer or an ulcer scar, because the involved mucosa cannot be separated sufficiently from the muscular layer by injecting saline solution into the submucosa. EMR specimens usually contain mucosa and the middle layer of the submucosa. The deep layer of the submucosa cannot be resected. Gastric cancer invading into or beyond the middle of the submucosa cannot be resected curatively and contraindicates EMR. Thus, early gastric cancer with an ulcer or an ulcer scar or invasion into the middle or deeper layer of the submucosa are contraindications to EMR.

With laser therapy, locally curative treatment for early gastric cancer can be obtained even when the lesion has fibrosis or submucosal invasion. The role of endoscopic laser therapy in patients with gastric cancer and advanced age or severe complications has been reported [11–13]. Endoscopic laser therapy has several advantages as a treatment for early gastric cancer in these cases.

First, endoscopic laser therapy is powerful and almost the full thickness of the submucosa can be irradiated depending on the laser output and the duration, so locally curative treatment can be obtained even when the lesion invades into the middle or deeper portion of the submucosa.

Second, endoscopic laser therapy rarely causes complications such as bleeding, perforation, or stenosis.

Table III Strategy of endoscopic treatment for early gastric cancer. (First Dept. of Surg. TMDU)

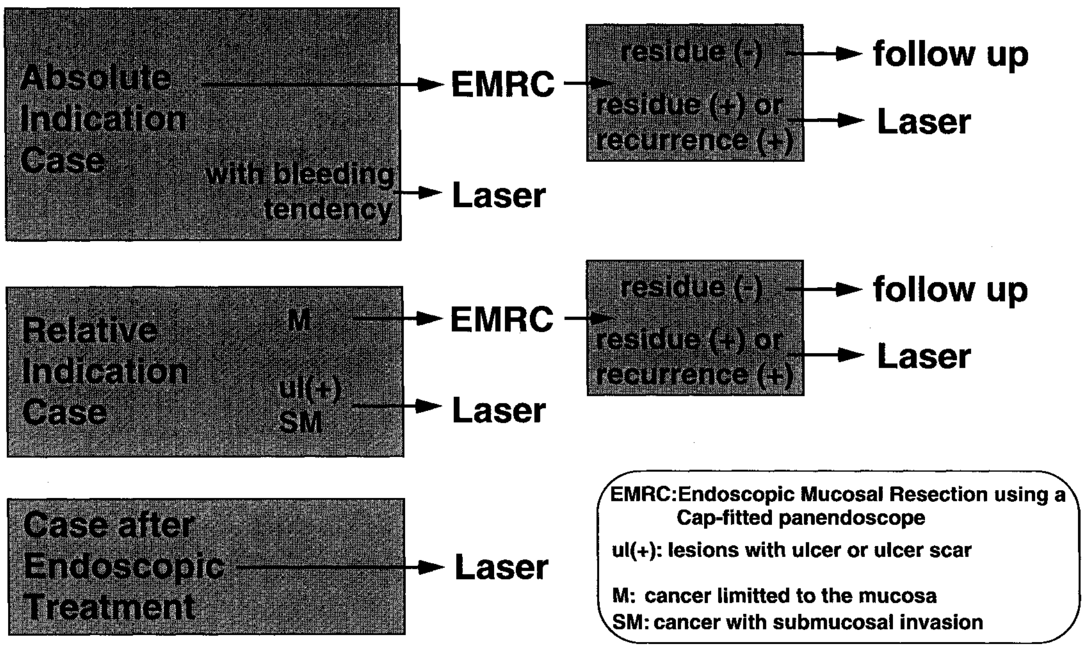

The overall complication rate of laser endoscopy in 1859 cases of gastrointestinal cancer in Tajiri's series was 2.9% [14]. The most frequent complication was bleeding, reported in 45 patients (2.4%), followed by perforation in 5 patients (0.3%) [14]. In our series we experienced no complications.

Third, laser therapy has a powerful hemostatic effect, and patients with clotting deficiencies can be safely treated.

Fourth, features of the medical YAG-laser system MC2100 make laser irradiationcan particularly safe and effective. Because 1.32-µm wavelength is absorbed by water ten times as much as the conventional 1.06-µm wavelength, effective irradiation to living tissue is available with lower output (10 to 30 watts compared to 50~70 watts respectively; see reference [15]).

Fifth, hospitalization is not necessary.

An EMRC cap is useful for diagnosis, resection, and hemostasis during EMR of gastric lesions. Using an EMRC cap, front views with adequate distance from the lesions in any location can be obtained, and the lesions can be measured relative to the diameter of the cap. Thus, precise assessment of lesions and accurate mucosal resection and hemostasis can be performed. In endoscopic laser therapy, an EMRC cap permits vertical irradiation with adequate distance between the laser fiber and the lesion.

One problem with endoscopic treatment is an inability to diagnose the stage of a lesion. It is particularly very difficult to diagnose the spread of undifferentiated adenocarcinoma. For those lesions, we perform four or more biopsies of normal mucosa surrounding the lesion and record the biopsy locations. During laser irradiation, we make marks surrounding the lesion based on the histological results of these biopsies, and irradiate an area of mucosa that encompasses all the marks. We hypothesise that this method will reduce the incidence of residual cancer.

In conclusion, we have found that endoscopic laser therapy can be performed on an outpatient basis. It can remove early gastric cancer even when the lesion includes an ulcer, scar, or submucosal invasion, and it has a powerful hemostatic effect. This therapy is a safe and effective treatment for early gastric cancer including lesions in poor surgical candidates and residual lesions following other endoscopic treatments.

### References

[1] Eckhauser, M.L. Palliative therapy of upper gastrointestinal malignancies using the Nd-YAG laser. *Am. Surg.*, 1990; **56**: 158–162.

[2] Brennan, F.N., Cowen, A.E., Laurence, B.H. Successful treatment of two patients with gastric antral vascular ectasia 'watermelon stomach' using endoscopic Nd-YAG laser therapy. *Aust. N.Z. J. Med.*, 1991; **21**: 439–441.

[3] Hiki, Y., Shimao, H., Mieno, H., Sakakibara, Y. Laser therapy for early upper gastrointestinal carcinoma. *Surg. Clin. North Am.*, 1992; **72**: 571–580.

[4] Inoue, H., Endo, M., Takeshita, K. *et al.* Endoscopic esophageal mucosal resection using a cap-fitted panendoscope (EMRC). *Gastroenterol. Endoscopy*, 1992; **34**: 2387–2390.

[5] Takeshita, K., Tani, M., Kando, F. *et al.* Results of endoscopic treatment in patients with early esophageal carcinoma or gastric cancer lesions: with special reference to the quality of the patient's life. (with English abstracts) *Progress of Digestive Endoscopy (Shoukaki Naisikyo No Shinpo)*, 1993; **44**: 57–62.

[6] Tani, M., Takeshita, K., Endo, M. *et al.* Endoscopic mucosal resection using a cap-fitted panendoscope (EMRC) for gastric tumorous lesion. (with English abstracts) *Progress of Digestive Endoscopy (Shoukaki Naisikyo No Shinpo)*, 1994; **44**: 73–76.

[7] Tani, M., Inoue, H., Kando, F. *et al.* Result of endoscopic treatment for early gastric cancer by EMRC and laser therapy. (with English abstracts) *Progress of Digestive Endoscopy (Shoukaki Naisikyo No Shinpo)*, 1995; **46**: 82–86.

[8] Japanese Research Society for Gastric Cancer Japanese classification of gastric carcinoma, first English edition Kanehara & CO., LTD. Japan, 1995.

[9] Ozaki, M., Urita, S., Ootsuka, Y., *et al.* Improved endoscopic therapies with a new transparent tip-hood. (with English abstracts) *Endoscopia Digestiva (Shoukaki Naishikyo)*, 1990; **2**: 659–666.

[10] Honda, T., Takeshita, K., Endo, M., *et al.* Indication of curative endoscopic therapy for early gastric cancer from the clinicopathological and endoscopic points of view. (with English abstracts) *Progress of Digestive Endoscopy (Shoukaki Naisikyo No Shinpo)*, 1993; **42**: 16–21.

[11] Tajiri, H., Oguro, Y. Laser endoscopic treatment for upper gastrointestinal cancers. *J. Laparoendosc. Surg.*, 1991; **1**: 71–78.

[12] Teixeira, C.R., Haruma, K., Teshima, H. *et al.* Endoscopic therapy for gastric cancer in patients more than 80 years old. *Am. J. Gastroenterol.*, 1991; **86**: 725–728.

[13] Korenaga, D., Watanabe, A., Saito, A., *et al.* Laser treatment for poor-risk patients with early gastric cancer: post treatment pathology. *Eur. J. Surg. Oncol.*, 1991; **17**: 316–318.

[14] Tajiri, H., Oguro, Y. Laser endoscopic treatment for upper gastrointestinal cancers. *Gann Monograph on Cancer Research*, 1990; **37**: 101–107.

[15] Shimao, H., Hiki, Y. A role of endoscopic laser treatment for early gastric cancer. (with English abstracts) *Progress of Digestive Endoscopy (Shoukaki Naisikyo No Shinpo)*, 1993; **42**: 38–42.

